# Does digital literacy affect the happiness of rural residents? Evidence from China

**DOI:** 10.3389/fpsyg.2025.1647907

**Published:** 2025-11-13

**Authors:** Yi Ding, Junnan Zhang

**Affiliations:** Vocational and Technical College, Inner Mongolia Agricultural University, Hohhot, China

**Keywords:** internet, CFPS, happiness, digital divide, digital literacy

## Abstract

**Introduction:**

Digital literacy is increasingly viewed as a prerequisite for psychological wellbeing in rural societies, yet evidence on its multidimensional effects remains fragmented.

**Methods:**

Drawing on a recent nationwide rural survey, we examined how distinct facets of digital literacy relate to residents’ happiness while accounting for potential endogeneity and heterogeneity across demographic groups. Robustness checks included instrumental-variable and propensity-score approaches; social trust and perceived income served as mediators.

**Results:**

Digital literacy showed a consistent positive association with subjective wellbeing that persisted across alternative specifications. The linkage was stronger among middle-aged and older adults, men, non-eastern regions, and less-educated individuals; digital work literacy exerted the largest influence. Social trust and perceived income partly explained the relationship.

**Discussion:**

Findings highlight digital literacy as a modifiable determinant of rural happiness and underscore the value of targeted, work-oriented digital-skill interventions for digitally vulnerable populations.

## 1 Introduction

In recent years, with the continuous development of digital technology and information platforms, China’s internet penetration rate and development quality have both seen significant improvements. According to the “China Internet Development Report 2024,” by the end of 2024, China’s internet user population had exceeded 1.1 billion, with an annual growth rate of over 7 million users, and the internet penetration rate had reached 78%. The rapid development of digital technology has brought about improvements in work efficiency and increased convenience in daily life (Wang X. et al., 2025; Wang S. et al., 2025). In rural areas, the internet has gradually played an important role in agricultural production, farmers’ daily lives, information search, and education and learning. Internet technology has brought new opportunities and conveniences to farmers’ lives and work. However, the digital benefits enjoyed by rural residents vary across different countries and regions, and this may be due to differences in digital literacy levels among rural residents. The concept of digital literacy originally referred to an individual’s willingness and ability to use internet-connected devices, leverage digital resources to enhance efficiency, or engage in interactions within a digital environment. Luan et al. (2025) demonstrate that the diffusion of Internet and digital technologies exerts a substantial influence on individuals’ welfare and day-to-day convenience. Complementing this finding, Wang X. et al. (2025) and Wang S. et al. (2025) shows that rural residents who acquire adequate digital literacy experience tangible gains in economic capacity and social rights. Taken together, these studies imply that raising the level of digital literacy is likely to be a pivotal lever for further improving the subjective wellbeing of China’s rural population.

Research on happiness has also sparked the interest of relevant scholars. To date, Dewinta et al. (2019) have conducted in-depth and detailed investigations at three levels: the micro-individual level, the meso-family level, and the macro-societal level. At the micro-individual level Meng et al. (2023a,b), found that differences in individual factors such as age, gender, education, and occupation may lead to variations in their levels of happiness. At the meso-level of the family, Pesci et al. (2023) argues that differences in family characteristics, such as family income level and family fixed assets, may lead to variations in individuals’ levels of happiness. At the macro-level of society, Abdullah (2015) and Zhao et al. (2023) suggests that differences in geographical regions or levels of environmental pollution may significantly impact individuals’ happiness, thereby leading to variations in their levels of happiness. Recently, researchers have begun to explore the connection between digital technology and rural residents’ happiness. For example, Hennessy et al. (2016) argues that the widespread application of digital technology can help reduce rural residents’ material desires, thereby playing an important role in enhancing their happiness levels. Additionally, Samadder et al. (2023) suggest that the application of digital technology and the internet can promote individual happiness levels by reducing information acquisition costs and increasing social activities. In-depth exploration of the factors and measures to improve residents’ wellbeing not only aligns with the governing principles of the Communist Party of China but also contributes to building a more harmonious and sustainable social environment.

The implementation of the “Digital China” strategy has substantially upgraded the nation’s digital infrastructure and fostered a deep integration of digital technologies into both production and daily-life scenarios. Nevertheless, the urban–rural developmental gap remains a fundamental national condition: rural residents face greater barriers to equitably sharing the dividends of digitalization and exhibit significantly lower digital literacy than their urban counterparts (Alnafrah and Belyaeva, 2024; Jun, 2021; Xie et al., 2021). Surprisingly, few studies have systematically examined the causal nexus between digital literacy and subjective happiness for this “digitally vulnerable” population, let alone the mechanisms through which any such effect might operate.

This paper makes three marginal contributions. First, the literatures on digital literacy and on residents’ happiness have remained largely disjoint. Rural Chinese residents are distinctive in that they are simultaneously confronted with intensifying human–land contradictions and with insufficient convergence between agricultural production and digital technologies. Evidence generated for this group therefore offers policy-relevant insights for optimizing digital-development strategies in China and other developing countries.

Second, we adopt a four-tier measurement framework—digital learning literacy, digital social literacy, digital work literacy and digital living literacy—and estimate the differential impacts of each dimension on farmers’ happiness, thereby furnishing a more granular analysis than has hitherto been available.

Third, we enrich the mechanistic evidence by demonstrating that social trust and subjective income perception mediate the effect of digital literacy on farmers’ happiness, unveiling new pathways through which the wellbeing of digitally disadvantaged groups can be improved.

## 2 Literature review

### 2.1 Factors and measurement of happiness

Subjective wellbeing (SWB) denotes an individual’s cognitive evaluation and affective response to his or her actual life circumstances; in the literature it is often used interchangeably with “happiness” and is conceived as a psychological, cognition-based reaction (Lu et al., 2023). Researchers typically use happiness, life satisfaction, and levels of joy as key indicators to measure an individual’s wellbeing. In previous studies, researchers primarily explored the levels of happiness from two perspectives: absolute and relative (Meng et al., 2023a,b; Wang and Dong, 2023). First, a representative view in studies from the absolute perspective is that an increase in absolute income will alleviate household budget constraints through an intermediary channel, thereby increasing an individual’s level of happiness (Wang X. et al., 2025; Wang S. et al., 2025; Meng et al., 2023a,b). An important premise of this research view is that the level of absolute income has not reached a bottleneck but can continue to grow steadily, thereby continuously easing an individual’s budget constraints. This increases their sense of attainment and utility realization, ultimately promoting their level of happiness to a certain extent. Building on this, researchers further investigated scenarios where absolute income levels have reached a plateau. According to the theory of relative deprivation, when individuals subjectively perceive that their circumstances in life, learning, and other contexts fail to meet their original expectations, they experience a sense of relative deprivation. Researchers found that this sense of deprivation also has a negative impact on individuals’ wellbeing. Therefore, an increase in relative income levels can help reduce individuals’ feelings of deprivation, thereby promoting the continuous growth of happiness levels. In recent years, as researchers have delved deeper into happiness studies, happiness has intersected with various other disciplines, becoming a hot topic in multiple research fields (Panganiban, 2018).

Subjective wellbeing (SWB) is typically measured either by a single global item or by multi-item scales. The single-item approach asks a concise evaluative question—e.g., “Overall, how happy have you felt recently?”—answered on 2- to 5-point or 0–10 numeric scales. This strategy is inexpensive, easy to administer, and readily comparable across surveys, yet it cannot disentangle affective from cognitive components and is sensitive to transient mood fluctuations. Scale-based instruments, such as the Satisfaction with Life Scale or PANAS, collect multiple ratings that are aggregated into a composite index through principal-component, entropy-weighting, or similar procedures. These instruments provide greater reliability and richer information on hedonic, eudaimonic and domain-specific evaluations, but they demand larger sample sizes, stricter item balancing, and more elaborate treatment of missing values; moreover, the resulting scores are difficult to compare over time or across cultures unless they are explicitly anchored. Researchers therefore trade off brevity and inter-temporal comparability against depth and internal consistency, selecting the approach that best serves their analytical objectives (Sari, 2023; Bontsa et al., 2023).

Additionally, Ji and Zhuang (2023) conducted a systematic and in-depth study on the factors influencing happiness. Researchers found that the factors currently capable of influencing happiness levels primarily concentrate on individual and external dimensions. At the individual level, these factors include personal income, personality traits, values, assets, and educational attainment, which form the foundation for assessing an individual’s happiness level and establish the framework for determining whether their happiness level is high or low (Hu and Wang, 2025). External factors, on the other hand, refer to factors other than the individual that influence happiness levels, such as economic conditions, unemployment rates, environmental quality, urban scale, and government policies. These factors also play a significant role in determining happiness levels (Rachmina et al., 2022). However, due to significant disparities between developed and developing countries in terms of national conditions, economic status, environmental conditions, and social security levels, most existing research conclusions are based on systematic studies centered on developed countries. These conclusions are not entirely applicable to developing countries, nor to China, which has even more complex national conditions. This also underscores the significant value and importance of conducting research centered on developing countries.

### 2.2 Research and measurement of digital literacy

As Internet penetration deepens, cloud platforms and agricultural big-data systems have become embedded in rural production routines and daily life, raising efficiency and incomes. Yet the welfare gains are uneven: individuals differ markedly in access, usage intensity and benefit capture, a phenomenon labelled the “digital divide” (Bai et al., 2023). The divide originates in digital literacy—the capacity to employ networked devices to acquire, integrate and create information—which is itself shaped by regional income levels, educational endowments and policy intensity. Contextual evidence shows that the marginal happiness gain from Internet use is larger for rural than for urban residents (Zhang and Zhang, 2024); however, inappropriate or excessive use can crowd out offline social participation and thereby reduce subjective wellbeing (Wang X. et al., 2025; Wang S. et al., 2025). Digital literacy therefore determines not only technological accessibility but also the ultimate welfare returns to digital engagement.

Regarding the measurement of digital literacy, different researchers have proposed varying measurement methods. Generally, researchers often use a multi-factor measurement approach to assess individuals’ digital literacy. For example, a series of questions reflecting the frequency and importance of internet use are posed, and the entropy weight method is employed to derive a unified variable for evaluating an individual’s digital literacy level (Magesa et al., 2023). Additionally, some researchers analyze the impact of digital literacy at different levels by categorizing dimensions such as internet usage frequency and perceived importance of the internet. Furthermore, other researchers evaluate an individual’s digital literacy level using concepts such as digital skills and the digital divide (Liu and Liao, 2024).

In summary, previous studies have employed relatively simple methods to measure digital literacy. Even when researchers have used multi-factor measurement methods to study individuals’ digital literacy, the lack of standardized evaluation criteria has led most researchers to assess this variable primarily through questions regarding internet usage frequency and perceived importance of the internet. This evaluation method fails to comprehensively and objectively measure digital literacy levels from multiple perspectives. Therefore, the conclusions drawn from previous measurement methods may not fully represent an individual’s digital literacy level.

### 2.3 The impact of digital literacy on happiness

A substantial body of work exists on both digital literacy and subjective happiness, yet the two strands remain only loosely connected; evidence from developing countries is particularly scarce. Most existing studies focus on high-income economies. Lee et al. (2022) report that Internet diffusion and smart-device adoption significantly raise voter turnout in national elections. Becker (2021) shows that digital media platforms expand the space for emotional expression and political opinion, thereby enhancing freedom of speech. Lu et al. (2023) find that higher digital literacy improves mental health, arguing that the effect is mediated by reduced relative deprivation. Conversely, Chen and Li (2025) warns that improper or excessive use of digital devices exposes individuals to misinformation, cyber-fraud and addictive behaviors, ultimately undermining emotional and psychological wellbeing.

Among the few studies set in developing countries, Wang et al. (2022) construct an information-based framework to analyze how digital literacy affects happiness, with particular attention to low-income farmers. Their analysis, however, is constrained by a small sample and the absence of dimension-specific estimates of digital literacy.

Overall, the literature has yet to reach a consensus on whether and how digital literacy influences happiness, and existing developing-country evidence is both dated and limited in coverage. Using the 2022 wave of the China Family Panel Studies (CFPS 2022), this paper examines the mechanisms linking digital literacy to the happiness of rural residents, documents heterogeneous impacts across multiple demographic and regional margins, and provides the first late-pandemic assessment of these relationships. The four-dimensional decomposition of digital literacy further enhances the policy relevance and practical value of our findings.

### 2.4 Research hypothesis

The literature has yet to reach a consensus on the linkage between digital literacy and happiness. Moderate engagement with digital devices tends to raise happiness (Wang and Qu, 2023), whereas excessive or improper use exerts the opposite effect (Li et al., 2025). Chinese rural residents confront a double challenge. On the material side, digitalization boosts productivity but simultaneously displaces low-skill jobs, imposing steeper skill requirements on groups already digitally disadvantaged. On the psychosocial side, low-cost online socializing exposes less-educated villagers to upward comparison, undermining life satisfaction.

Recent rural digital-inclusion policies in China have notably alleviated these risks. Infrastructure expansion paired with targeted training has upgraded residents’ digital skills, expanded off-farm employment channels and raised incomes; stringent content regulation ensures that online information is predominantly positive, curbing harmful exposure (Li et al., 2023). Drawing on the extended technology-acceptance model, we contend that such policy interventions act as external variables that lower access barriers and enhance perceived usefulness and ease of use, thereby amplifying the positive effect of digital literacy on happiness. We therefore expect the net impact of improved digital literacy to be positive and propose:

*H1*: An increase in rural residents’ digital literacy significantly raises their happiness.

Regarding mechanisms, we invoke social-capital theory and argue that digital literacy accelerates the accumulation of social capital, thereby strengthening generalized social trust and raising happiness (Du et al., 2024). Higher digital literacy enables villagers to use relatively sophisticated services—government portals, village WeChat groups, short-video platforms, etc. Because China’s cyberspace is subject to strict content review, the information encountered online is largely positive, pro-social and stability-oriented. Repeated exposure to such curated content reduces social uncertainty and signals governmental competence and benevolence, fostering both institutional and interpersonal trust. Higher trust lowers psychological transaction costs, strengthens “sense of gain” and social integration, and ultimately translates into greater happiness. We therefore propose:

*H2*: Digital literacy raises rural residents’ happiness by enhancing social trust.

In addition, relative-deprivation theory suggests that digital literacy improves perceived income status by shifting the reference group downward, thereby increasing happiness. As digital literacy rises, rural residents gain access—via e-commerce platforms, short-video apps and live-streaming—to a much wider socioeconomic spectrum (Soo and Yena, 2020). Exposure to visibly worse-off individuals weakens relative deprivation, generates psychological superiority and elevates subjective income rank. Because utility evaluations are reference-dependent, this upward revision of subjective socioeconomic status raises satisfaction and, consequently, happiness. We therefore propose:

*H3*: Digital literacy raises rural residents’ happiness by improving perceived income status.

**Figure 1:**
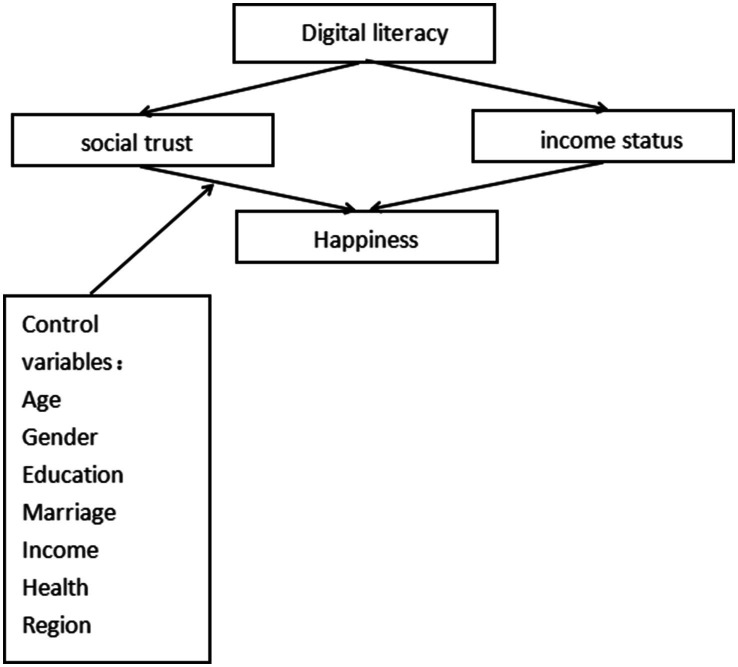
Research framework.

Figure 1 summarizes the study logic and framework.

## 3 Materials and methods

### 3.1 Data

The data used in this study comes from a nationwide, large-scale, comprehensive survey project, the China Family Panel Studies (CFPS), conducted in 2022. This is also the latest round of the CFPS survey, which effectively reflects the current state of digital literacy and happiness among rural residents in China, among other research-related topics. The CFPS 2022 primarily employs a combination of multistage equal probability sampling and implicit stratified sampling in its sampling methodology. Specifically, the first step involves selecting administrative district-level terminal sampling units as the target population. Subsequently, based on these administrative district-level terminal sampling units, administrative villages are chosen as the secondary sampling units. Finally, the scope of the terminal sampling units is further narrowed down to households and individuals. To ensure the reliability of the sampling results, CFPS primarily used a random-start cyclic sampling method to select the sampling objects. The age range of the selected research subjects was primarily 16–80 years old, providing a relatively comprehensive representation of the development of Chinese society, families, the economy, and agriculture.

Before conducting empirical analysis, the author performed necessary data cleaning to ensure the reliability of the research conclusions. First, since the research subjects of this paper were rural residents in China, only samples with rural household registration locations were retained. Second, samples with missing independent variables, dependent variables, and control variables were excluded. Finally, after completing the data cleaning process, a total of 2,085 valid samples that met the requirements were obtained.

### 3.2 Variables

#### 3.2.1 Dependent variable: happiness

In this study, the CFPS2022 research questionnaire was used, which includes a single question to measure the happiness levels of rural residents in China. The questionnaire asks, “How happy are you?.” The options range from 0 to 10, with higher scores indicating higher levels of happiness.

#### 3.2.2 Independent variable: digital literacy

In this study, the measurement standards for digital literacy proposed by Zhao et al. (2022) were primarily referenced. Specifically, digital literacy was broken down into four aspects—digital learning literacy, digital social literacy, digital work literacy, and digital life literacy—for separate evaluation. Ultimately, principal component analysis (PCA) was used to standardize the data, resulting in variables capable of evaluating the overall level of digital literacy among rural residents in China. Specifically, digital learning literacy is primarily measured using two questions: “Do you use the internet for learning?” and “How important is the internet for learning?” Digital social literacy is primarily measured using two questions: “How frequently do you share content on social media?” and “How important is maintaining contact with family and friends?” Digital work literacy is primarily measured using two questions: “Do you use WeChat for work?” and “How important is the internet for work?” Digital life literacy is measured through five questions: “Do you play online games?” “Do you shop online?” “Do you watch short videos?” “How important is the internet for leisure and entertainment?” and “How important is the internet for daily life?” The specific options and scoring principles are detailed in the descriptive statistics.

#### 3.2.3 Control variables

Following the research by Du et al. (2024), the following control variables were selected for this study.

Age. The questionnaire asked, “What is your age?.” Respondents answered based on their actual age, and the variable “age” was constructed accordingly.

Gender. The questionnaire asked, “What is your gender?” A binary variable “gender" was created based on the responses, with a value of 1 for male and 0 for female.

Education. The questionnaire asked, “What is your highest level of education?” The response options included illiterate, elementary school, junior high school, high school/vocational school/technical school, college, undergraduate, graduate, and above. Based on the options, a binary variable “education” was created, with a value of 0 for high school/vocational school/technical school education or below, and a value of 1 for high school/vocational school/technical school education or above.

Income. The questionnaire asks, “What was your household income in the past year?” Based on the response, the variable “income” is created, with the specific income amount from the response assigned as the variable value.

Marital status. The questionnaire asks, “What is your current marital status?” Based on the response, a binary dummy variable “marital status” is created, with a value of 0 assigned when the response is “unmarried,” and a value of 1 assigned otherwise, indicating “married.” This is used to measure marital status.

Health. The questionnaire asks, “How would you rate your health?” A variable “health” is created based on the response, with options ranging from 1 to 5. The higher the score, the higher the self-rated health level.

Region. To control for the impact of different regions on the study, two binary dummy variables, region 1 and region 2, are constructed to represent the respondent’s regional location. All regions are divided into eastern and western categories with the central region as the boundary, and this is reflected through region 1 and region 2.

Political affiliation. The questionnaire asks, “Are you a member of the Communist Party of China?” Based on the response, a binary variable “party” is created, with a value of 1 assigned when the answer is yes and a value of 0 assigned when the answer is no.

#### 3.2.4 Mechanism variables

Referring to the research findings of Yang et al. (2021) and the perspectives of relative deprivation theory, this paper selects social trust level, subjective income perception, and subjective social status perception as the mechanism variables for this study. The study explores the impact of digital literacy on the happiness of rural residents in China.

Regarding social trust levels, the questionnaire item “Do you prefer to trust others or doubt others?” is used for measurement, and a binary variable “trust” is created based on the responses. There are two options available: when choosing to doubt others more, the value is set to 0; when answering that one prefers to trust others more, the value is set to 1.

Regarding subjective income perception, the questionnaire item “Where do you think your income ranks locally?” was used for measurement, and a variable “income” was created based on the responses. The response options ranged from 1 to 5, with higher values indicating a higher level of subjective income perception among respondents.

Regarding subjective social status perception, the questionnaire item “Where do you think your social status stands locally?” is used for measurement, and a variable “status” is created based on the response. The response options range from 1 to 5, with higher values indicating a higher level of subjective social status perception among respondents.

Descriptive statistics are presented in Table 1.

**Table 1:** Descriptive statistics.

Project	Option	Count	*N*	Percentage
Digital learning literacy				
Do you use online learning	Yes = 1	290	2,085	13.91
	No = 0	1795	2,085	86.09
How important is the internet for learning	Very unimportant = 1	177	2,085	8.49
	Unimportant = 2	184	2,085	8.82
	Somewhat important = 3	489	2,085	23.45
	Important = 4	447	2,085	21.44
	Very important = 5	788	2,085	37.79
Digital social literacy				
Frequency of sharing on social media	Never = 1	840	2,085	40.29
	Once every few months = 2	470	2,085	22.54
	Once a month = 3	251	2,085	12.04
	2–3 times a month = 4	220	2,085	10.55
	1–2 times a week = 5	204	2,085	9.78
	3–4 times a week = 6	47	2,085	2.25
	Almost daily = 7	53	2,085	2.54
How important is it for you to stay in touch with family and friends	Not at all important = 1	32	2085	1.53
	Not important = 2	66	2,085	3.17
	Somewhat important = 3	231	2,085	11.08
	Important = 4	427	2,085	20.48
	Very important = 5	1,329	2,085	63.74
Digital work literacy				
Do you use WeChat for work	Yes = 1	1994	2,085	95.64
	No = 0	91	2,085	4.36
Importance of the Internet for work	Very unimportant = 1	213	2,085	10.22
	Unimportant = 2	176	2,085	8.44
	Moderately important = 3	544	2,085	26.09
	Important = 4	351	2,085	16.83
	Very important = 5	801	2,085	38.42
Digital literacy				
Do you play online games	Yes = 1	308	2,085	14.77
	No = 0	1777	2,085	85.23
Do you shop online	Yes = 1	825	2,085	39.57
	No = 0	1,260	2,085	60.43
Do you watch short videos	Yes = 1	1,436	2085	31.13
	No = 0	649	2,085	68.87
Importance of the internet for leisure and entertainment	Very unimportant = 1	154	2,085	7.39
	Unimportant = 2	226	2,085	10.84
	Somewhat important = 3	690	2,085	33.09
	Important = 4	425	2,085	20.38
	Very important = 5	590	2,085	28.30
Importance of the internet for daily life	Very unimportant = 1	209	2,085	10.02
	Unimportant = 2	227	2,085	10.89
	Somewhat important = 3	445	2,085	21.34
	Important = 4	390	2,085	18.71
	Very important = 5	814	2,085	39.04
Control variables				
Gender	Male = 1	1,120	2,085	53.72
	Female = 0	965	2,085	46.28
Education	High school or above = 1	413	2,085	19.81
	High school or below = 0	1,672	2,085	80.19
Marital status	Married = 1	1,690	2,085	81.06
	Unmarried = 0	395	2,085	18.94
Health	Very poor = 1	270	2,085	12.95
	Poor = 2	150	2,085	7.19
	Average = 3	931	2,085	44.65
	Good = 4	364	2,085	17.46
	Excellent = 5	370	2,085	17.75
Region1	Located in the eastern region = 1	670	2,085	32.13
	Not located in the eastern region = 0	1,415	2,085	67.87
Region2	Located in the western region = 1	1,082	2,085	51.89
	Not located in the western region = 0	1,003	2,085	48.11
Party	Yes = 1	24	2,085	1.15
	No = 0	2061	2,085	98.85

Project	Count	Average	Median	Minimum	Maximum	Standard deviation	Kurtosis	Skewness
Happiness	2,085	7.21	8	0	10	2.15	3.27	−0.60
Digital literacy	2,085	0	0.21	−5.34	3.79	1.76	2.73	−0.47
Age	2,085	43.24	43	16	80	13.41	2.15	0.02
Income	2,085	96923.32	70	0	3,500,000	153413.2	259.7009	13.31

### 3.3 Research methodology

This paper uses the OLS model to conduct an in-depth study of the impact of digital literacy on the happiness of rural residents in China. The reason for this is that the OLS model can control the influence of other variables when analyzing the impact of an independent variable on a dependent variable, a feature that meets the requirements of this paper’s in-depth study of the impact of digital literacy on the happiness of Chinese farmers. Referring to Gao (2022) research method, the model is constructed as follows:


$$\begin{array}{l} \;\;\\ \text{Happiness}=\alpha 0+\text{αnDigital}+\alpha 2n\;X+\mathrm{\Sigma i} \end{array}$$ (1)

In (1), Happiness represents the level of happiness among rural residents in China and serves as the dependent variable in this study. Digital represents the level of digital literacy among rural residents in China and serves as the independent variable in this study. X refers to a series of control variables, including age, gender, income, education, marital status, health, region1, region2, and party affiliation. α0 represents the constant term, while αn and α2n represent the core parameters to be estimated. Σi denotes the random disturbance term.

Mediation-effect model. To dissect the mechanisms through which digital literacy influences the happiness of rural Chinese residents, we test the mediating roles of social trust and perceived income status. Following Alvarez-Bartolo and MacKinnon (2024), we augment Equation 1 with a three-step procedure:


Digital=β0+βnTrust+β2nX+Σi (2)

Digital=γ0+γnincome+γ2nX+Σi (3)

Happiness=λ0+λnDigital+λ2nMediating+λ3nX+Σi (4)

In Equations 2–4, Happiness is defined as in Equation 1 and denotes the level of happiness of rural Chinese residents. Digital represents the level of digital literacy among rural Chinese residents. Mediating refers to the two mediating variables, namely, the level of social trust and the perception of subjective income. *β*₀, *γ*₀, and *λ*₀ denote the constant terms. βₙ, γₙ, λₙ, β₂ₙ, γ₂ₙ, λ₂ₙand λ3ₙ represent the regression coefficients. X represents a set of control variables, such as gender, age, and marital status. εᵢ represents the random disturbance term. In the process of testing whether the mediating effect is established, the stepwise regression method is chosen. First, the significant effect of the independent variable Digital on the dependent variable Happiness is verified. Then, the significant effect of the mediating variables on the independent variable is examined. Finally, both the independent variable Digital and the mediating variables are included to re-estimate the effect on the dependent variable Happiness. If the regression results are significant and the regression coefficient of the last test decreases, the mediating effect is considered to be established.

## 4 Empirical analysis

### 4.1 Model testing

This paper employs the OLS model to investigate the impact of digital literacy on the happiness of rural residents in China. To ensure the reliability of the research conclusions and the appropriateness of the model selection, this paper will conduct multicollinearity and autocorrelation tests on the model. The primary purpose of multicollinearity testing is to ensure the independence of the independent variables in regression analysis, thereby enhancing the accuracy and stability of regression coefficient estimates, and ultimately improving the model’s explanatory and predictive capabilities. This paper uses the VIF test method for multicollinearity testing. The autocorrelation test helps ensure the independence of the model assumptions. By conducting an autocorrelation test, the accuracy and reliability of the model selection can be ensured, and the predictive accuracy can be further improved. This study uses the Corr test to examine the autocorrelation of the model. The results of the VIF test and Corr test are shown in Table 2. Part 2a of Table 2 shows the results of the multicollinearity test. Generally, when the VIF value is less than 5, it indicates that the multicollinearity issue is not severe, while a VIF value greater than 5 indicates a more severe multicollinearity issue. The VIF value for the independent variable Digital is 1.17, the 1/VIF value is 0.85, and the mean VIF is 1.35, indicating that digital has almost no multicollinearity issues. The VIF values for the other control variables are also all less than 5, which eliminates the impact of multicollinearity on the research conclusions, ensuring the reliability of the research findings. Part 2b of Table 2 shows the results of the autocorrelation test. According to the test results, the correlation between most variables is relatively weak, but there are some variables with relatively strong correlations, such as the correlation coefficient between digital and age, which is −0.3244, indicating that the older the age, the lower the frequency of digital technology use, which aligns with normal patterns. However, overall, there are no severe autocorrelation issues between most variables that could affect the reliability of the research conclusions. Therefore, the model selection and variable selection are relatively appropriate.

**Table 2:** Model testing.

(a) Multicollinearity test (VIF)		
Variable	VIF	1/VIF
Digital	1.17	0.85
Age	1.59	0.62
Gender	1.04	0.96
Edu	1.11	0.89
Income	1.02	0.97
Mar	1.36	0.73
Health	1.08	0.92
Region1	2.07	0.48
Region2	2.09	0.47
Party	1.01	0.99
Mean VIF	1.35	

(b) Autocorrelation test (corr)										
Variable	Digital	Age	Gender	Edu	Fincome	Mar	Health	Region1	Region2	Party
Digital	1.0000									
Age	−0.3244	1.0000								
Gender	0.0182	0.0513	1.0000							
Edu	0.2245	−0.2411	0.0317	1.0000						
Income	0.0884	−0.0550	−0.0158	0.0550	1.0000					
Mar	−0.1137	0.4889	−0.0928	−0.1712	0.0334	1.0000				
Health	0.1522	−0.2548	0.0583	0.0663	0.0257	−0.1211	1.0000			
Region 1	0.0006	0.0874	−0.0575	−0.0508	0.0411	0.0496	0.0205	1.0000		
Region 2	−0.0041	−0.1035	0.0458	−0.0249	−0.0836	−0.0637	0.0073	−0.7147	1.0000	
Party	0.0220	−0.0536	0.0280	0.0479	0.0008	−0.0052	−0.0066	−0.0069	0.0049	1.0000

### 4.2 Benchmark testing

Building on the previous chapter, we estimate the effect of digital literacy on the happiness of rural Chinese residents with OLS. Multicollinearity and autocorrelation diagnostics confirm that the specification is statistically sound. The digital-literacy index is standardized via PCA. Eleven underlying indicators yield a KMO statistic of 0.81 and a Bartlett test *p* < 0.001, indicating suitability for factor reduction. One component (eigenvalue = 3.12) is retained, explaining 58.3% of the total variance.

Table 3 reports the results. Without controls, the coefficient on digital literacy is 0.222; with the full set of covariates it is 0.224. Both estimates are positive and significant at the 1% level, indicating that higher digital literacy robustly raises rural residents’ happiness. Hypothesis H1 is therefore supported. Detailed interpretation of the control variables is omitted, as they are not the focus of this study.

**Table 3:** Benchmark test results.

Variable	(1)	(2)
Happiness		
Digital	0.222*** (8.45)	0.224*** (8.16)
Age		0.00731* (1.73)
Gender		−0.0468 (−0.51)
Edu		−0.0815 (−0.69)
Mar		0.553*** (4.14)
Income		−0.000000487 (−1.64)
Health		0.351*** (8.97)
Region1		0.305** (2.20)
Region2		−0.177 (−1.36)
Party		0.880** (2.08)
*N*	2,085	2,085
*R* ^2^	0.0331	0.0939
Control variables	NO	YES

① *, **, and *** denote significance at the 10, 5, and 1% levels, respectively. ② t-statistics are reported in parentheses.

### 4.3 Robustness test

To ensure the reliability of the research conclusions, this paper conducted a series of robustness tests, including five tests: replacing the independent variable, replacing the dependent variable, replacing the research model, trimming the top and bottom 5% of age levels, and replacing the control variables. The specific test results are shown in Table 4.

**Table 4:** Robustness test.

A robustness test 1: replacing the independent variable	(1)	(2)
Happiness		
Divide	−0.131*** (−4.22)	−0.144*** (−4.19)
*N*	2,085	2,085
*R* ^2^	0.0085	0.0727
Control variables	NO	YES

B robustness test 2: replacing the dependent variable	(1)	(2)
Happiness		
Digital	0.0989*** (8.42)	0.0961*** (7.81)
*N*	2,085	2,085
*R* ^2^	0.0329	0.0925
Control variables	NO	YES

C robustness test 3: replacing the econometric model	(1)	(2)
Happiness		
Digital	0.030* (0.004)	0.031*** (0.004)
*N*	2,085	2,085
Wald χ^2^	67.08***	215.79***
Pseudo *R*^2^	0.008	0.027
Control variables	NO	YES

D robustness test 4: truncated at 5%	(1)	(2)
Happiness		
Digital	0.222*** (8.45)	0.224*** (8.15)
*N*	2,085	2,085
*R* ^2^	0.0331	0.0939
Control variables	NO	YES

E robustness test 5: replacing control variables	(1)	(2)
Happiness		
Digital	0.222*** (8.45)	0.206*** (7.34)
*N*	2,085	2,085
*R* ^2^	0.0331	0.0937
Control variables	NO	YES

① *, **, and *** denote significance at the 10, 5, and 1% levels, respectively. ② t-statistics are reported in parentheses.

Robustness Test 1: Replacing the core independent variable. To ensure that the estimated effect of digital literacy is not an artefact of a particular operationalization, we substitute the digital divide index. Following Choi et al. (2023), the index combines access divide (no mobile or computer Internet) and usage divide (no engagement in online gaming, shopping, short video viewing or e-learning) into five binary indicators. PCA yields KMO = 0.69, Bartlett χ^2^(15) = 3,509, *p* < 0.001. The first component (eigenvalue = 2.58) is retained, explaining 53.0 percent of the variance; all loadings are positive and exceed 0.48. The resulting Divide index is standardized so that higher values indicate a wider divide. Panel A of Table 4 shows that Divide significantly reduces happiness (*β* = −0.131 without controls, *β* = −0.144 with controls, both *p* < 0.01), confirming that the baseline finding—digital literacy raises rural Chinese residents’ happiness—is robust to alternative measurement.

Robustness Test 2: Replacing the Dependent Variable. To ensure the reliability of the study, this paper replaced the scoring criteria for the dependent variable Happiness and used Happiness’ for regression analysis. When Happiness equals 1 or 2, Happiness’ is assigned a value of 1; when Happiness equals 3 or 4, Happiness’ is assigned a value of 2; when Happiness equals 5 or 6, Happiness’ is assigned a value of 3. When Happiness equals 7 or 8, Happiness’ is assigned a value of 4. When Happiness equals 1 or 2, Happiness’ is assigned a value of 5. The specific results are shown in Part B of Table 4. When control variables are not controlled, the regression coefficient is 0.0989. When control variables are controlled, the regression coefficient is 0.0961. Regardless of whether control variables are considered, the coefficient remains significantly positive at the 1% level, thereby ensuring the robustness of the benchmark test conclusions.

Robustness Test 3: Alternative estimator. To ensure that the positive effect of digital literacy on happiness is not an artefact of the OLS specification, we follow Wang et al. (2022) and re-estimate the model with an ordered Probit that explicitly accounts for the 0–10 ordered discrete nature of the dependent variable. Panel C of Table 4 presents the results. Column (3) reports the average marginal effect (AME) for the highest happiness category. With or without controls, the digital-literacy coefficient remains positive and significant, and the Wald χ^2^ statistic is highly significant, confirming that the baseline finding is robust to the choice of estimator.

Robustness Test 4: 5% Winsorization. To mitigate the influence of extreme values, we winsorize the top and bottom 5% of the age distribution. Panel D of Table 4 shows that, with or without controls, the estimated coefficients on digital literacy are 0.222 and 0.224, respectively, and remain significant at the 1% level—virtually identical to the baseline estimates—confirming that our findings are not driven by outliers.

Robustness Test 5: Alternative controls. Drawing on the study by Lu et al. (2023), First, we add two durable-asset indicators—home ownership (1 = own, 0 = otherwise) and private-car ownership (1 = own, 0 = otherwise). Following wealth-stratification theory, these assets signal long-run economic capacity and shape social comparison; controlling for them isolates the pure effect of digital skills from the prestige channel. If digital literacy still raises happiness when housing and car ownership are held constant, the gain is more plausibly attributed to genuine capability enhancement than to unobserved wealth. Second, we recode the education variable into six ordered categories: below upper-secondary = 1, upper-secondary/technical = 2, associate degree = 3, bachelor = 4, master = 5, doctorate = 6, and replace the original regional dummies with a simple east versus non-east split. Panel E of Table 4 shows that the coefficient on digital literacy is 0.206 (*p* < 0.01) after these adjustments—direction and significance unchanged, magnitude only slightly lower than the baseline 0.224—confirming that the core finding survives controls for visible wealth and alternative measurement schemes.

In summary, whether replacing the independent variable, replacing the dependent variable, replacing the econometric model, tailing, or replacing the control variable, the benchmark research conclusion that digital literacy has a significant positive impact on the happiness of rural residents in China remains robust, and a series of robustness tests can also ensure the reliability of the research conclusions.

### 4.4 Endogeneity test

To establish a credible causal effect of digital literacy on the happiness of rural Chinese residents and minimize systematic bias, we address endogeneity that stems from three sources: measurement error, omitted variables, and simultaneity. Measurement error is mitigated ex ante through multicollinearity and autocorrelation diagnostics; omitted-variable bias is attenuated by an extensive set of controls; simultaneity is tackled with an instrumental-variable (IV) design. Following Shin et al. (2025) and Lu et al. (2023), we use the leave-one-out, same-sex village mean of digital literacy as the instrument. Peer interactions transmit operational digital knowledge (e.g., app usage, anti-fraud tips) rather than resources that directly determine happiness, ensuring relevance. Village fixed effects absorb all community-level public goods (roads, base stations, clinics, etc.), and individual-level controls (income, education, marital status, etc.) sever any direct pathway from the instrument to respondents’ happiness, satisfying the exclusion restriction.

Table 5 reports two-stage least-squares (2SLS) estimates. In the first stage, the instrument carries coefficients of 1.459 without controls and 1.413 with controls (both *p* < 0.01). Cragg–Donald Wald F statistics are 1,685.5 and 1,199.0, far above the Stock–Yogo 10% threshold of 16.4, rejecting weak instruments. Hausman tests yield χ^2^ = 0.77 (*p* = 0.38) and χ^2^ = 0.11 (*p* = 0.74), indicating no significant endogeneity bias. Hence, the baseline OLS estimates are reliable.

**Table 5:** Endogeneity test.

Variable	(1)	(2)
Happiness		
Digital	0.196*** (5.00)	0.212*** (4.67)
Age		0.00683 (1.53)
Gender		−0.0453 (−0.49)
Edu		−0.0732 (−0.60)
Mar		0.557*** (4.15)
Fincome		−0.000000479 (−1.61)
Health		0.352*** (8.96)
Region1		0.306** (2.21)
Region2		−0.177 (−1.36)
Party		0.879** (2.08)
First-stage regression coefficients	1.459*** (41.05)	1.413*** (34.63)
*R* ^2^	0.0327	0.4564
Hausman test value	0.77	0.11
*P*-value	0.3804	0.7391
F-statistic	1685.5	1199.0
Observations	2,085	2,085
Control variables	No	Yes

① *, **, and *** denote significance at the 10, 5, and 1% levels, respectively. ② t-statistics are reported in parentheses.

### 4.5 Dimensional disaggregation test

To examine whether the four dimensions of digital literacy exert differential effects on farmers’ subjective wellbeing, we conducted separate regressions after PCA standardization of each subscale; results are reported in columns (1)–(4) of Table 6. All subscales satisfy the minimum requirements for factor analysis. Digital learning literacy: KMO = 0.50, Bartlett χ^2^ = 79.8, *p* < 0.001, eigenvalue = 1.19, variance explained = 59.7%. Digital social literacy: KMO = 0.55, χ^2^ = 15.6, *p* < 0.001, eigenvalue = 1.09, variance explained = 64.3%. Digital work literacy: KMO = 0.56, χ^2^ = 11.4, p < 0.001, eigenvalue = 1.07, variance explained = 63.7%. Digital life literacy: KMO = 0.58, χ^2^ = 1138.2, *p* < 0.001, eigenvalue = 1.83, variance explained = 60.6%.

**Table 6:** Dimensional disaggregation test.

Variable	(1)	(2)	(3)	(4)
Digital learning literacy	0.215*** (4.40)			
Digital social literacy		0.288*** (6.23)		
Digital work literacy			0.736*** (16.34)	
Digital life literacy				0.159** (3.06)
*N*	2,085	2,085	2,085	2,085
*R* ^2^	0.0735	0.0821	0.1716	0.0691
Control variables	YES	YES	YES	YES

① *, **, and *** denote significance at the 10, 5, and 1% levels, respectively. ② t-statistics are reported in parentheses.

The estimates reveal a clear hierarchy. Digital work literacy yields the largest happiness gain (*β* = 0.736, *t* = 16.34), followed by digital social literacy (*β* = 0.288) and digital learning literacy (*β* = 0.215), whereas digital life literacy has the smallest effect (*β* = 0.159).

This ordering is consistent with the JD-R model. Skills that directly expand work related resources such as online search, mobile payment and e commerce tools deliver the highest marginal utility to rural residents who rely heavily on farming and off farm employment (Demerouti et al., 2001). Work oriented digital resources raise productivity and income while also satisfying basic psychological needs for competence and autonomy, thereby generating the strongest wellbeing returns. By contrast, leisure oriented digital activities such as online games and short videos operate mainly through hedonic adaptation; although they provide immediate pleasure, the affective boost is transient and rapidly habituates, resulting in a smaller and less durable impact on life satisfaction.

Thus, although all dimensions of digital literacy are beneficial, policies that prioritize work-related digital skills are likely to generate the greatest subjective wellbeing dividends for rural Chinese residents.

### 4.6 Heterogeneity test

To examine how the happiness effect of digital literacy varies across subgroups of rural Chinese residents, we conduct heterogeneity analyses along four dimensions proposed by Gutiérrez et al. (2024): age, gender, region and education. These tests not only fill an academic gap but also provide policy guidance for improving rural residents’ digital literacy and thereby raising their happiness. Detailed results are reported in Table 7.

**Table 7:** Heterogeneity test.

(a) Heterogeneity test 1: age				
Variable	Age ≤45		Age>45	
Happiness				
Digital	0.223*** (5.84)	0.217*** (5.65)	0.262*** (6.67)	0.241*** (6.11)
Count	1,146	1,146	939	939
*R* ^2^	0.0289	0.0966	0.0453	0.1106
Control variable	No	Yes	No	Yes

(b) Heterogeneity test 2: gender				
Variable	Male		Female	
Happiness				
Digital	0.247*** (6.77)	0.239*** (6.49)	0.195*** (5.14)	0.208*** (4.98)
Count	1,120	1,120	965	965
*R* ^2^	0.0394	0.1222	0.0267	0.0756
Control variable	No	Yes	No	Yes

(c) Heterogeneity test 3: region				
Variable	Eastern region		Non-eastern region	
Happiness				
Digital	0.192*** (4.59)	0.211*** (4.86)	0.237*** (7.17)	0.230*** (6.53)
Count	670	670	1,415	1,415
*R* ^2^	0.0305	0.1157	0.0352	0.0754
Control variable	No	Yes	No	Yes

(D) Heterogeneity test 4: education	High school or below		High school or above	
Happiness				
Digital	0.220*** (7.28)	0.206*** (6.71)	0.287*** (4.91)	0.337*** (5.42)
Count	1,672	1,672	413	413
*R* ^2^	0.0307	0.1004	0.0553	0.0986
Control variable	No	Yes	No	Yes

① *, **, and *** denote significance at the 10, 5, and 1% levels, respectively. ② t-statistics are reported in parentheses.

Age heterogeneity: using the WHO cut off of 45 years, we split the sample into youth (≤45) and middle aged/older (>45) groups. Panel A shows that both coefficients are positive and significant at the 1% level, but the point estimate is larger for the older group (0.241 with controls) than for the youth group (0.217), indicating that digital literacy yields a significantly stronger happiness premium among middle aged and older villagers. This pattern is plausible because older residents face fewer leisure and social options, so digital devices that reduce loneliness and broaden information channels deliver higher marginal utility.

Gender heterogeneity: panel B presents separate estimates for men and women. Both coefficients are significant at 1%, yet the magnitude is consistently larger for men (0.239 with controls) than for women (0.208), showing that the positive effect is significantly stronger for male villagers. In rural China men typically bear greater income and employment pressures; improved digital literacy raises their productivity and job opportunities, thereby boosting happiness more markedly.

Region heterogeneity: we divide the sample into eastern (*N* = 670) and non-eastern (*N* = 1,415) regions. Panel C reveals that although both sub samples display significant positive effects at the 1% level, the coefficient is larger in non-eastern areas (0.230 with controls) than in eastern areas (0.211). The result aligns with relative deprivation theory: because digital infrastructure and economic opportunities are scarcer in non-eastern regions, the same increase in digital ability yields a larger reduction in deprivation and hence a larger happiness gain.

Education heterogeneity: panel D reports estimates split by senior high school completion. Digital literacy is positive and significant (*p* < 0.01) in both groups, yet the coefficient is noticeably larger for respondents with at least a senior high education (0.337) than for those without (0.220). The higher estimate suggests that digital literacy enhances happiness more among better educated rural residents, probably because they use digital technologies in a wider range of work and life contexts, so higher literacy opens up more and better job opportunities and conveniences, thereby raising wellbeing more substantially.

### 4.7 Mediation mechanism test

To identify the channels through which digital literacy raises the happiness of rural Chinese residents, we estimate a parallel mediation model following Ding et al. (2025). Social trust and perceived income are specified as simultaneous mediators; all equations use robust standard errors and the full set of covariates. Table 8 summarizes the three-step results: columns (1)–(3) correspond to the Baron-Kenny sequence for each mediator.

**Table 8:** Mechanism of action tests.

(A) Social trust			
Item	(1)	(2)	(3)
Variable	Happiness	Trust	Happiness
Digital	0.224*** (8.16)	0.0743*** (5.52)	0.194*** (7.15)
*N*	2,085	2,085	2,085
*R* ^2^	0.0939	0.0802	0.1300
Indirect effect (bootstrap)			0.033 [0.004, 0.072]***
Control variables	Yes	Yes	Yes

(B) Perceived income			
Item	(1)	(2)	(3)
Variable	Happiness	Income	Happiness
Digital	0.224*** (8.16)	0.0743*** (5.52)	0.194*** (7.15)
*N*	2,085	2,085	2,085
*R* ^2^	0.0939	0.0802	0.1300
Indirect effect (bootstrap)			0.038 [0.022, 0.062]***
Control variables	Yes	Yes	Yes

① *, **, and *** denote significance at the 10, 5, and 1% levels, respectively. ② t-statistics are reported in parentheses.

Social trust. Panel A shows that digital literacy significantly raises generalized trust (*β* = 0.074, *t* = 5.52, *p* < 0.01). When trust is added to the happiness equation the direct effect of digital literacy falls from 0.224 to 0.194 while remaining highly significant. Bias-corrected bootstrapping (5,000 replications) yields a significant indirect effect [*β* = 0.033, 95% CI (0.004, 0.072)], confirming partial mediation. The finding supports the virtuous-circle argument: stronger digital skills increase exposure to positively curated information, elevate trust, reduce relative deprivation and thereby improve subjective happiness.

Perceived income. Panel B repeats the procedure for perceived income. Digital literacy significantly enhances perceived income (*β* = 0.074, *t* = 5.52, *p* < 0.01); controlling for this perception reduces the direct happiness effect from 0.224 to 0.194. The bootstrapped indirect path is again significant [*β* = 0.038, 95% CI (0.022, 0.062)], indicating that digital literacy raises happiness by convincing individuals that their financial situation is better—a mechanism consistent with the relative-deprivation hypothesis.

Taken together, the results corroborate Hypotheses H2 and H3: digital literacy boosts happiness not only directly but also indirectly through higher social trust and improved perceived income. Policymakers should therefore bundle digital-skills programs with interventions that strengthen community trust and make income gains more visible, thereby maximizing wellbeing gains for rural residents.

## 5 Discussion

The rapid development of the Internet has actively driven changes in Chinese society, the economy and daily life. In contemporary society, the Internet and digital technologies have penetrated every aspect of how Chinese people learn, live and work and exert an important influence, while the level of digital literacy is the most direct reflection of an individual’s mastery of digital technology (Polizzi, 2021). In recent years, society has increasingly emphasized cultivating individuals’ digital literacy so that they can adapt to a rapidly developing society, enjoy the advantages brought by the digital dividend and further improve their living standards (Bansal and Choudhary, 2024). Chinese rural residents are an extremely special group that currently faces the contradiction between low digital literacy and the rapid development of a digital society. Because education and Internet penetration are uneven, rural residents have not been able to fully enjoy the social benefits brought by the rapid development of digital technology (Bhatia and Ritchie, 2016). The existence of this situation has become a problem that must be addressed in order to promote the overall growth of Chinese people’s happiness, yet research on this issue is relatively scarce (Zhao X. et al., 2024; Zhao B. et al., 2024). Most studies either treat all residents as the research object or focus only on urban residents, and existing studies on rural residents mostly concentrate on Internet technology or the digital divide; few researchers have conducted in-depth studies on the concept of digital literacy itself. The difference between digital literacy and Internet technology is that digital literacy is essentially a deeper manifestation of Internet technology, encompassing digital learning literacy, digital social literacy, digital work literacy and digital life literacy, providing a more comprehensive, complete and reasonable evaluation of rural residents’ Internet technology, and the conclusions obtained through this approach are more targeted and complete. Therefore, this study not only supplements the existing literature on digital literacy and happiness but also reveals the phenomenon of happiness loss among digital vulnerable groups such as farmers when they face digital inequality, which has important practical significance and value (Kim et al., 2025). It encourages researchers and policymakers to include digital vulnerable groups in the scope of policy dividends when dealing with the allocation of digital resources, ultimately promoting wider access to the convenience and wellbeing brought by digital technology development.

## 6 Policy recommendations

The findings of this study carry significant international value. At present, research on digital literacy remains markedly uneven between developed and developing countries (Kusumastuti and Nuryani, 2020). Our results therefore not only offer China’s grassroots authorities practical, evidence-based guidance, but also provide a reference for other developing countries designing policies to raise rural residents’ digital literacy and, in turn, their happiness (Liu and Zhou, 2023). In the long run, the study contributes to global policy debates and embodies a humanitarian commitment to ensuring that digital dividends reach the most disadvantaged populations.

Targeting policy by heterogeneity: Empirical results show that the happiness return to digital literacy is significantly higher for men (*β* = 0.239) than for women (*β* = 0.208) and for residents older than 45 years (*β* = 0.241) than for younger adults (*β* = 0.217). Training quotas and subsidies should therefore be explicitly weighted toward these two groups. For rural men, the income-safety channel dominates: subjective income perception accounts for 0.038 of the total happiness gain. Bundling digital-literacy modules with e-commerce micro-credit—such as Ant Group’s “Village Manager Loan” pilot—can amplify wellbeing without additional fiscal outlays. For middle-aged and older villagers, the benefit flows mainly from increased social trust and reduced loneliness [indirect effect = 0.033, 95% CI (0.004, 0.072)]. Digital-social training courses that improve their ability to use social platforms will expand online interaction and alleviate isolation. Concurrent campaigns that teach verification of online information and healthy device use can further raise trust and, consequently, happiness (Kerkhoff and Makubuya, 2021). Heterogeneity results further reveal that the happiness gain from digital literacy is larger in eastern than in non-eastern regions (0.237 versus 0.192 without controls; 0.230 versus 0.211 with controls). Infrastructure investment should therefore be re-oriented toward non-eastern areas, expanding both the coverage and quality of base stations and fiber-optic networks to narrow the digital-divide gap. Supply-side outlays must be coupled with demand-side measures (Ji et al., 2024). Targeted training and policy support for local industrial digitalization can raise digital work literacy among non-eastern farmers, improve job conditions and earnings, and thereby enhance happiness.

Beyond China, the heterogeneous patterns documented here offer immediate guidance for other low- and middle-income countries that confront comparable rural digital divides. The elasticity of wellbeing with respect to digital literacy is significantly larger among older and male cohorts—groups that typically face higher income risk and greater social isolation. Uniform training curricula therefore risk misallocating scarce development funds. Embedding age- and gender-specific modules within existing social-protection or agricultural-extension platforms can generate larger happiness gains without increasing fiscal outlays. Likewise, the finding that marginal benefits are greatest where digital infrastructure is weakest implies that infrastructure subsidies should be spatially sequenced: less-developed regions should receive priority before any nationwide scale-up; otherwise early gains will accrue mainly to counties that already enjoy superior connectivity, thereby widening intra-rural inequality (Yongqi Zhang, 2023). The large agricultural populations, pronounced gender gaps and uneven regional development characteristic of these countries mirror the Chinese context analyzed here, so our empirical results remain highly relevant for their policy design.

## 7 Research prospects

This paper conducts an in-depth study on the impact of digital literacy on the wellbeing of rural residents in China, followed by a thorough discussion, ensuring the reliability of the research conclusions. As the internet continues to develop, digital technology plays an increasingly important role in people’s lives and has become an indispensable tool for the development of various industries. However, the uneven pace of internet development has left vulnerable groups such as rural residents unable to fully benefit from the advantages and conveniences brought by the digital dividend. The findings of this study demonstrate that effectively enhancing the digital literacy of China’s rural residents in the future will significantly improve their levels of wellbeing. This not only supports the Chinese government’s “Agricultural Powerhouse” policy but also contributes to social stability, sustained economic growth, and improved living standards for rural residents, thereby holding significant importance and value. However, the development of the internet in China has been characterized by rapidity and uncertainty. Therefore, in the future, it is essential to continue conducting in-depth research on the impact of digital literacy on the wellbeing and other aspects of rural residents, with the aim of improving their quality of life and achieving comprehensive benefits.

## 8 Limitations

Although this study adopts a rigorous design that includes endogeneity and robustness checks, several limitations remain. First, the analysis relies on the latest wave of the China Family Panel Studies (CFPS), which captures the impact of digital literacy on rural residents’ happiness in the late-pandemic period but cannot reveal long-term dynamics. Future research should exploit panel data to trace these effects over time. Second, despite the inclusion of an extensive set of controls and the use of an instrumental variable to mitigate endogeneity, some simultaneity bias may persist. Subsequent studies could search for more convincing instruments or incorporate additional controls to further reduce such bias. Notwithstanding these constraints, the methodological approach and detailed discussion offered here continue to provide valuable insights for both academics and policymakers.

## 9 Conclusion

This study employs the latest wave of data from the China Family Panel Studies (CFPS) conducted by Peking University in 2022, focusing specifically on rural Chinese residents. Below are the detailed conclusions of our research.

First, we conducted tests for model and variable selection, including multicollinearity and autocorrelation diagnostics. The results indicate that our model and selected variables are free from severe multicollinearity and autocorrelation issues, making them suitable for this study. Second, we performed baseline estimations, examining the impact of digital literacy on the happiness of rural residents both with and without control variables. In both cases, digital literacy exhibits a significant positive effect on happiness.

To ensure the reliability of our findings, we conducted a series of robustness checks. Additionally, to address potential endogeneity concerns, we employed an instrumental variable (IV) approach. The results confirm that our study does not suffer from significant endogeneity bias, thus reinforcing the robustness of our conclusions.

Dimensional analysis reveals that digital work literacy has the most substantial impact on happiness, while digital life literacy has the least. Furthermore, we conducted heterogeneity tests to explore how the impact of digital literacy on happiness varies across different subgroups defined by age, gender, region, and education. The results show that the positive effect of digital literacy on happiness is more pronounced among middle-aged individuals, males, residents of non-eastern regions, and those with higher education levels.

Regarding mechanism tests, we used a stepwise regression approach to examine the pathways through which digital literacy affects happiness. The results indicate that both social trust and subjective income perception play mediating roles in the relationship between digital literacy and happiness.

## Data Availability

The datasets presented in this study can be found in online repositories. The names of the repository/repositories and accession number(s) can be found in the article/supplementary material.
